# Tap Test Can Predict Cognitive Improvement in Patients With iNPH—Results From the Multicenter Prospective Studies SINPHONI-1 and –2

**DOI:** 10.3389/fneur.2021.769216

**Published:** 2021-11-02

**Authors:** Madoka Nakajima, Shigeki Yamada, Masakazu Miyajima, Kaito Kawamura, Chihiro Akiba, Hiroaki Kazui, Etsuro Mori, Masatsune Ishikawa, The SINPHONI-2 Investigators, Masaaki Hashimoto

**Affiliations:** ^1^Department of Neurosurgery, Juntendo University Faculty of Medicine, Tokyo, Japan; ^2^Department of Neurosurgery, Shiga University of Medical Science, Otsu, Japan; ^3^Department of Neurosurgery, Koto Geriatric Medical Center, Juntendo University School of Medicine, Tokyo, Japan; ^4^Department of Neuropsychiatry, Kochi Medical School, Kochi University, Nankoku, Japan; ^5^Department of Behavioral Neurology and Neuropsychiatry, Osaka University United Graduate School of Child Development, Suita, Japan; ^6^Normal Pressure Hydrocephalus Center, Rakuwakai Otowa Hospital, Kyoto, Japan

**Keywords:** cerebrospinal fluid shunt, normal pressure hydrocephalus, healthy life expectancy, cognitive function, Mini-Mental State Examination

## Abstract

**Background:** We analyzed the predictive value of the tap test (TT) on the outcome of cerebrospinal fluid (CSF) shunting in patients with idiopathic normal pressure hydrocephalus (iNPH) and cognitive impairment up to 12 months postoperatively.

**Methods:** We analyzed the data of two prospective multicenter studies on ventriculoperitoneal shunt (VPS) and lumboperitoneal shunt (LPS) use in iNPH patients. We selected patients with Mini-Mental State Examination (MMSE) scores ≤ 26 points as study subjects. We used a multivariate logistic regression model to obtain the optimal threshold of MMSE scores after TT to predict the score improvement at 12 months following shunting and that helped to control for confounding factors such as age and MMSE scores before TT. We used logistic regression models to identify variables with age-adjusted odds ratio (A-OR) and multivariate-adjusted OR (M-OR).

**Results:** For an improvement of ≥3 points in the MMSE score cutoff 7 days following TT in VPS and LPS cohort studies, the MMSE scores improved by 6 points after 12 months. The VPS cohort had sensitivity, specificity, and area under the curve (AUC) of 69.2, 73.7, and 0.771%, respectively; however, for the LPS cohort, they were 86.2, 90.9, and 0.906%, respectively. For MMSE scores that improved by ≥3 points in patients after the TT, the possibility of an improvement by 6 points at 12 months following CSF shunt had A-OR 7.77 and M-OR 6.3 times for the VPS, and A-OR 62.3 and M-OR 59.6 times for the LPS cohort.

**Conclusion:** CSF shunting contributes to improved cognitive function in iNPH patients. Furthermore, MMSE score evaluation at the TT can sensitively predict improvement in postoperative MMSE scores following LPS intervention.

**Clinical Trial Registration:** SINPHONI-1 (ClinicalTrials.gov, no. NCT00221091), first posted: September 22, 2005.

SINPHONI-2 [University Hospital Medical Information Network (UMIN) Clinical Trials no. UMIN000002730], the posted: February 1, 2010.

## Introduction

Idiopathic normal pressure hydrocephalus (iNPH) is a syndrome that occurs in older adults presenting with enlarged cerebral ventricles during diagnostic imaging and neurological symptoms, including gait disturbance with trunk balance disorder, cognitive impairment, and urinary incontinence (1–3). A cerebrospinal (CSF) shunt generally leads to an improvement in symptoms. The diagnosis and management of iNPH has progressed since the establishment of the current guidelines (4–10).

The rate of cognitive impairment in iNPH patients varies widely (11). A decline in cognitive function is important since it is the most burdensome symptom from the perspective of a caregiver (12). Some cohort studies have reported that improvement in cognitive function occurs slightly later than gait improvement (13). However, most of these were subjective evaluations (14). Global cognitive status previously assessed by Mini-Mental State Examination (MMSE) baseline scores is the widely applied quantitative predictor of cognitive outcomes (15–17). Although iNPH appears as a treatable form of dementia, an improvement in cognitive function has not been verified. In 1985, Black reported that shunting may not lead to improvement if dementia occurred first or is a major symptom (18). The perception that cognitive function in patients with iNPH does not meaningfully improve is apparently persisting (19).

In a multicenter, prospective cohort study of iNPH on neurological improvements (SINPHONI)-1, shunting was routinely performed on all enrolled patients, without judging the indications of surgery according to the CSF tap test (TT), which involves the removal of 30–50 ml of CSF (20, 21). Patients who improved by one or more parameters on a modified Rankin Scale (mRS), which evaluates the activities of daily living, were classified as shunt responders (SR). In a previous SINPHONI-1 study, the ventriculoperitoneal shunt (VPS) intervention was used, and it was reported that 69% of the patients were SR, thus, showing improvement in one or more parameters on the mRS after 12 months. SINPHONI-2 reported improvement in 61% of the cases with a lumboperitoneal shunt (LPS) intervention (22, 23). Nonetheless, an improvement in MMSE scores was demonstrated even in groups classified as non-SRs when evaluated with mRS (14, 24). Furthermore, the extent of change in MMSE scores after TT to predict the change after shunt intervention is not yet known. Moreover, with regard to treatment methods for patients with iNPH, researchers do not know if VPS or LPS should be prioritized from the perspective of cognitive function.

We aimed to analyze the predictive value of the TT for the effects of CSF shunting on patients with iNPH and cognitive impairment up to 12 months postoperatively, from the multicenter collaborative studies SINPHONI-1 and SINPHONI-2, involving two different shunting techniques.

## Methods

### Study Population

We retrospectively analyzed the prospectively collected cognitive outcomes from SINPHONI-1 (ClinicalTrials.gov, no. NCT00221091) and SINPHONI-2 [University Hospital Medical Information Network (UMIN) Clinical Trials no. UMIN000002730] patient cohorts. The recruitment and methodology of these studies have been described previously (20, 22, 23, 25).

**Figure 1:**
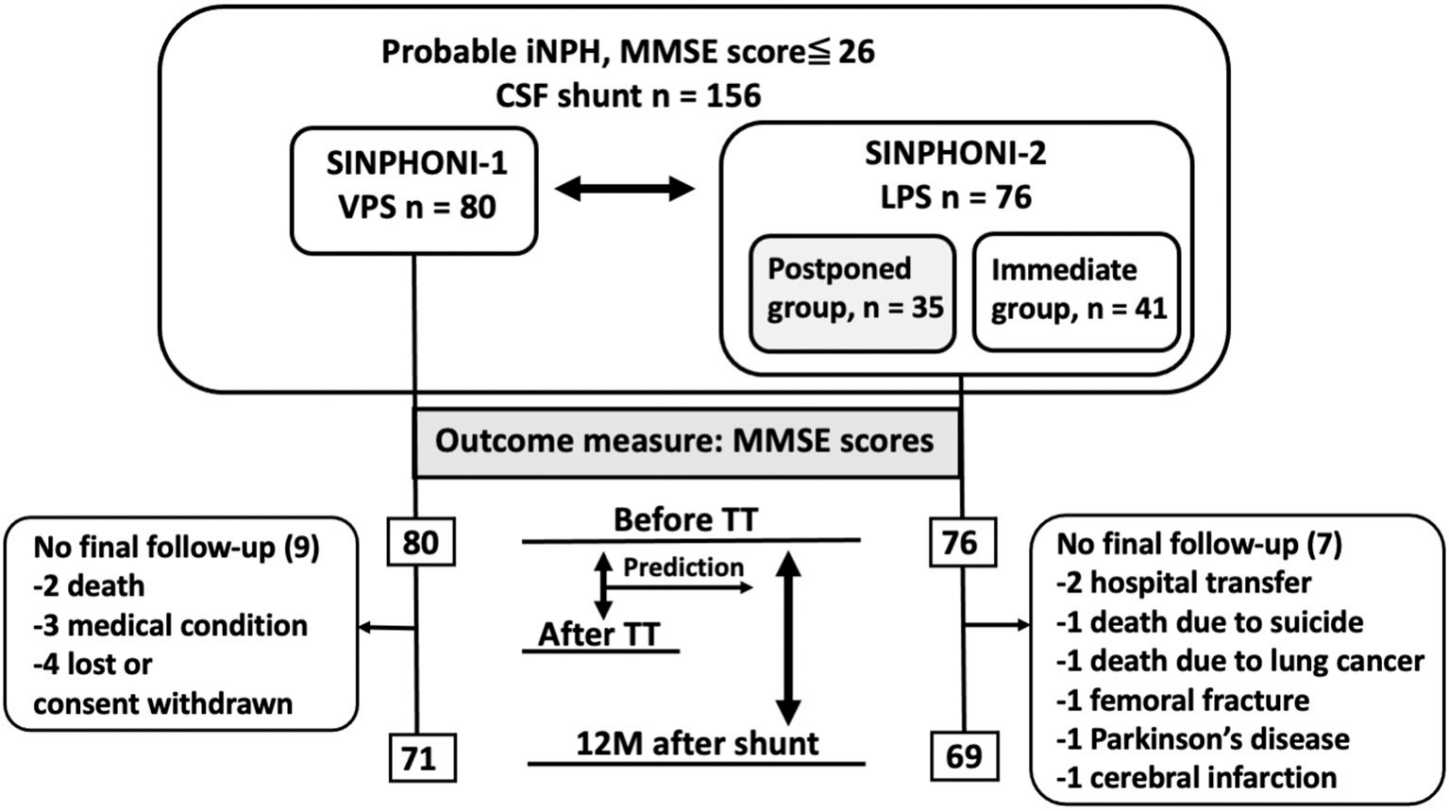
Flow chart displaying patients with probable iNPH and MMSE scores ≤26 before TT. CSF, cerebrospinal fluid; LPS, lumboperitoneal shunt; MMSE, Mini-Mental State Examination; iNPH, idiopathic normal pressure hydrocephalus; TT, tap test; VPS, ventriculoperitoneal shunt.

**Figure 2:**
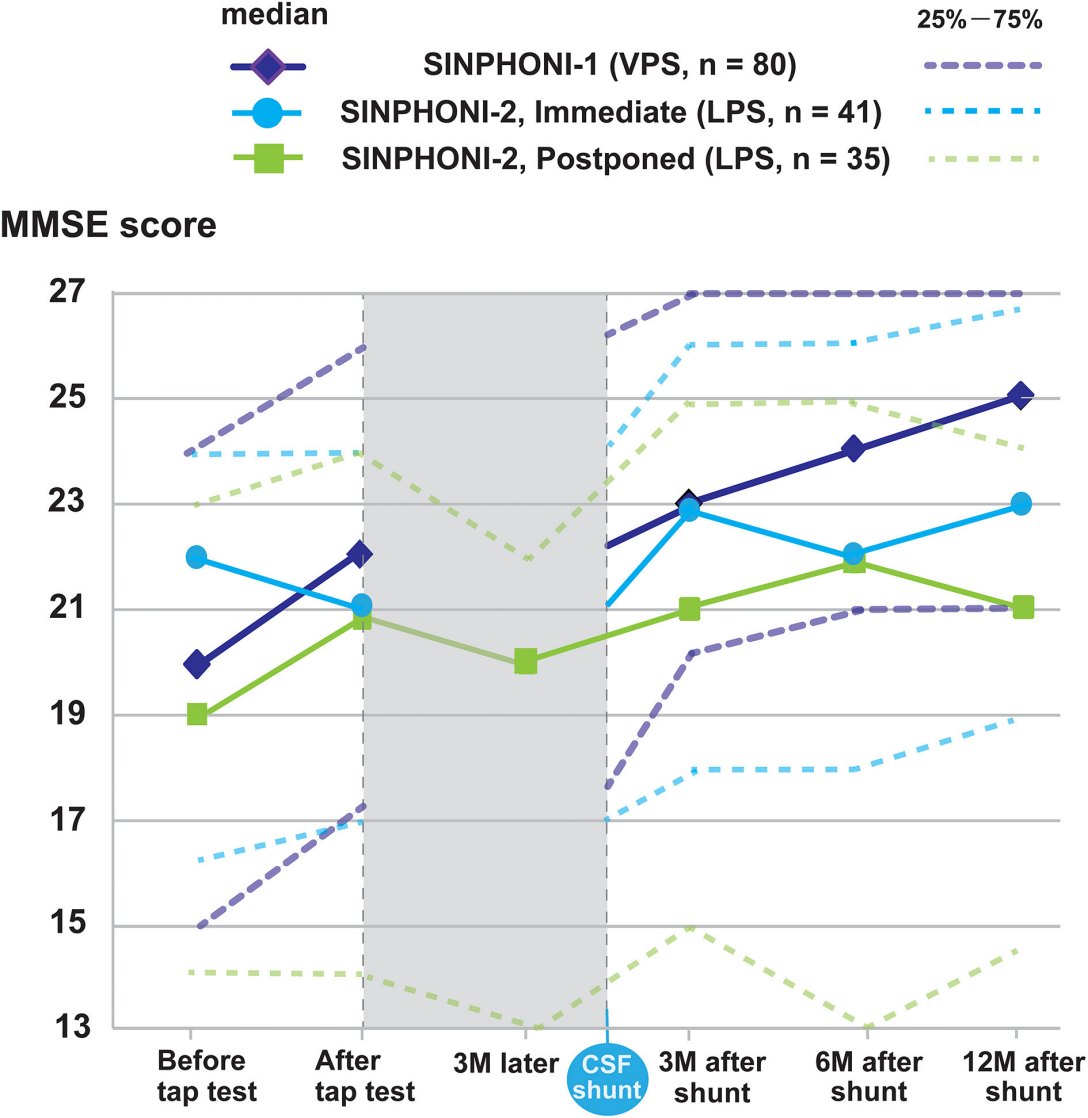
Changes in average MMSE scores for subjects with an MMSE score ≤26 during research participation. The changes in MMSE score of the cohort are shown as median MMSE score (solid line), 25–75% (dashed line). In SINPHONI-1, the median MMSE score (25–75%) are 20 (15–24) before tap test (TT), 22 (17–26) after TT, 23 (20–27) at 3 months, 24 (21–27) at 6 months, and 25 (21–27) at 12 months after VPS, respectively. For the immediate group in SINPHONI-2, the median MMSE scores are 22 (16.5–24) before TT, 21 (17–24) after TT, 23 (18–26) at 3, 6 months, and 23 (19–26.75) at 12 months after LPS, respectively. For the postpone group in SINPHONI-2, the median MMSE scores are 19 (14–23) before TT, 21 (14–24) after TT, 20 (13–22) at 3 months later, 21 (15–25) at 3 months, 22 (13–25) at 6 months, and 21 (14–24) at 12 months after VPS, respectively. CSF, cerebrospinal fluid; LPS, lumboperitoneal shunt; MMSE, Mini-Mental State Examination; VPS, ventriculoperitoneal shunt; SINPHONI, a multicenter, prospective cohort study of iNPH on neurological improvements; TT, tap test.

We selected patients with probable iNPH and cognitive impairment, including mild cognitive impairment (MCI) and an MMSE score ≤26 points as study subjects. A total of 156 subjects were classified as follows (Figure 1): (i) SINPHONI-1 group [*n* = 80; age: median, interquartile range (IQR, 25–75%), 75 (72–78) years] who underwent a TT and VPS and (ii) SINPHONI-2 group [*n* = 76; age 77 (73–79.75) years]. Patients in the latter group included a postponed group [*n* = 35, 77 (72.75–80) years] who underwent shunting after 3 months of waiting and an immediate group [*n* = 41, 77 (73–79) years] who underwent LPS at an early stage (Figure 2). There was no significant difference in the MMSE scores of the postponed group before TT [20, (14–23)] and after 3 months of waiting [before shunting, 20 (15.25–23)] (Wilcoxon's signed rank test, p = 0.673). Therefore, the two SINPHONI-2 groups, the immediate and postponed groups were combined to form one LPS group.

We evaluated the MMSE scores before TT, 7 days after TT, and 12 months postoperatively (SINPHONI-1, *n* = 71; SINPHONI-2, *n* = 69).

### Outcome Measures

A CSF TT consisted of drawing out ≥30 ml of CSF via a lumbar tap. Physical therapists, independent of the neurosurgeons performing the CSF shunt surgeries, recorded the clinical symptoms before and 7 days after TT and during the postsurgical follow-up points (12 months later). Cognition was evaluated using MMSE scores 7 days following TT (21, 26), as the change in cognitive function after the tap test, as assessed by Matsuoka et al. in the Montreal Cognitive Assessment scores, was more pronounced after 7 days (13).

We evaluated the degree of improvement by dividing the group of patients with cognitive impairment by three points on the MMSE score and dividing them by the severity of their illness. In the first study, the severity was classified into four groups using the MMSE scores before surgery as follows: grade (G)1, 24–26 points, MCI; G2, 21–23 points, mild dementia; G3, 18–20 points, moderate dementia; and G4, 0–17 points, severe dementia. MMSE scores alone were used as an indicator of the outcome. Patients with iNPH and MMSE scores that improved to ≥27 points following the TT or postoperatively were classified as G0. The degree of change in MMSE scores after the TT and 12 months following the shunt intervention were classified as one of the following four levels: excellent improvement (two ranks up from the severity group classification at entry), improvement (one rank up), no change, and worse (one or more ranks down).

Other cognitive function tests such as frontal assessment battery (FAB) scores, symbol search subtest of the Wechsler Adult Intelligence Scale—Third Edition (WAIS-III), Trail Making Test (TMT)-A (sec.), and Zarit Caregiver Burden Interview (ZBI) were assessed before and 12 months following shunting (Supplementary Tables 1, 2).

In the second study, we analyzed the optimal threshold, sensitivity, and specificity for MMSE score change after the TT for predicting MMSE score improvements at 12 months following shunting for SINPHONI-1 and SINPHONI-2.

To assess the predicted outcomes 12 months after the shunt surgery, we applied two definitions of MMSE score improvement: (i) ≥3 points or (ii) ≥6 points.

### Statistical Analyses

We used a per protocol set for all efficacy analyses, which made the data set compatible with those of SINPHONI-1 and SINPHONI-2. We performed the Wilcoxon's signed-rank test to analyze the median values and interquartile range (IQR, 25–75%) for age, MMSE, FAB, WAIS-III, TMT-A, and ZBI. The relationship of MMSE scores before TT to after TT and after CSF shunt was examined using Spearman's rank correlation coefficients.

We plotted the receiver-operating characteristic curve (ROC) of CSF TT for predicting the shunt effectiveness. Furthermore, we examined the association of improvement in MMSE scores following TT and after shunting to elucidate the reasons behind differences in accuracy of TT between SINPHONI-1 and SINPHONI-2. We used logistic regression models to search for variables with age-adjusted odds ratio (A-OR) and multivariate-adjusted OR (M-OR). A multivariate logistic regression model was used to control for confounding factors, such as age and MMSE scores before TT.

The odds ratios, 95% confidence intervals (CIs), and the probability (*p*) values of Fisher's exact test were calculated. Statistical significance was considered at a *p*-value < 0.05. All missing data were treated as deficit data that did not affect other variables. All statistical analyses were performed using SPSS version 25 (SPSS, Cary, NC, USA) for Windows and R software (version 3.0.1; R Foundation for Statistical Computing, Vienna, Austria); http://www.Rproject.org).

### Standard Protocol Approvals, Registrations, and Patient Consents

In SINPHONI-1 (ClinicalTrials.gov, no. NCT00221091), the patients were enrolled between November 2004 and November 2006. The Translational Research Informatics Center (TRI-Kobe, Japan) together with the steering committee, monitored all clinical data, imaging data, data related to safety issues, and protocol compliance via a web-based case report system. In SINPHONI-2 (University Hospital Medical Information Network Clinical Trials, no. UMIN000002730), patients were recruited from 20 Japanese institutes and hospitals between March 2010 and October 2011, and was designed by all the SINPHONI-2 investigators including a biostatistician and Independent Data and Safety Monitoring Committee members, in conformity with the Guidelines for Good Clinical Practice and the Declaration of Helsinki of the World Medical Association. The institutional review boards for the SINPHONI-1 and−2 studies approved the study protocol. All patients or their representatives provided written informed consent. All clinical and radiological data were prospectively recorded in an independent protocol compliance center via a web-based case report system. An authorization has been obtained for information disclosure (consent to disclose) for publication in a journal, in derivative works by the AAN, or on a journal's website.

## Results

### Changes in Average Mini-Mental State Examination Scores

#### Tap Test

**Figure 3:**
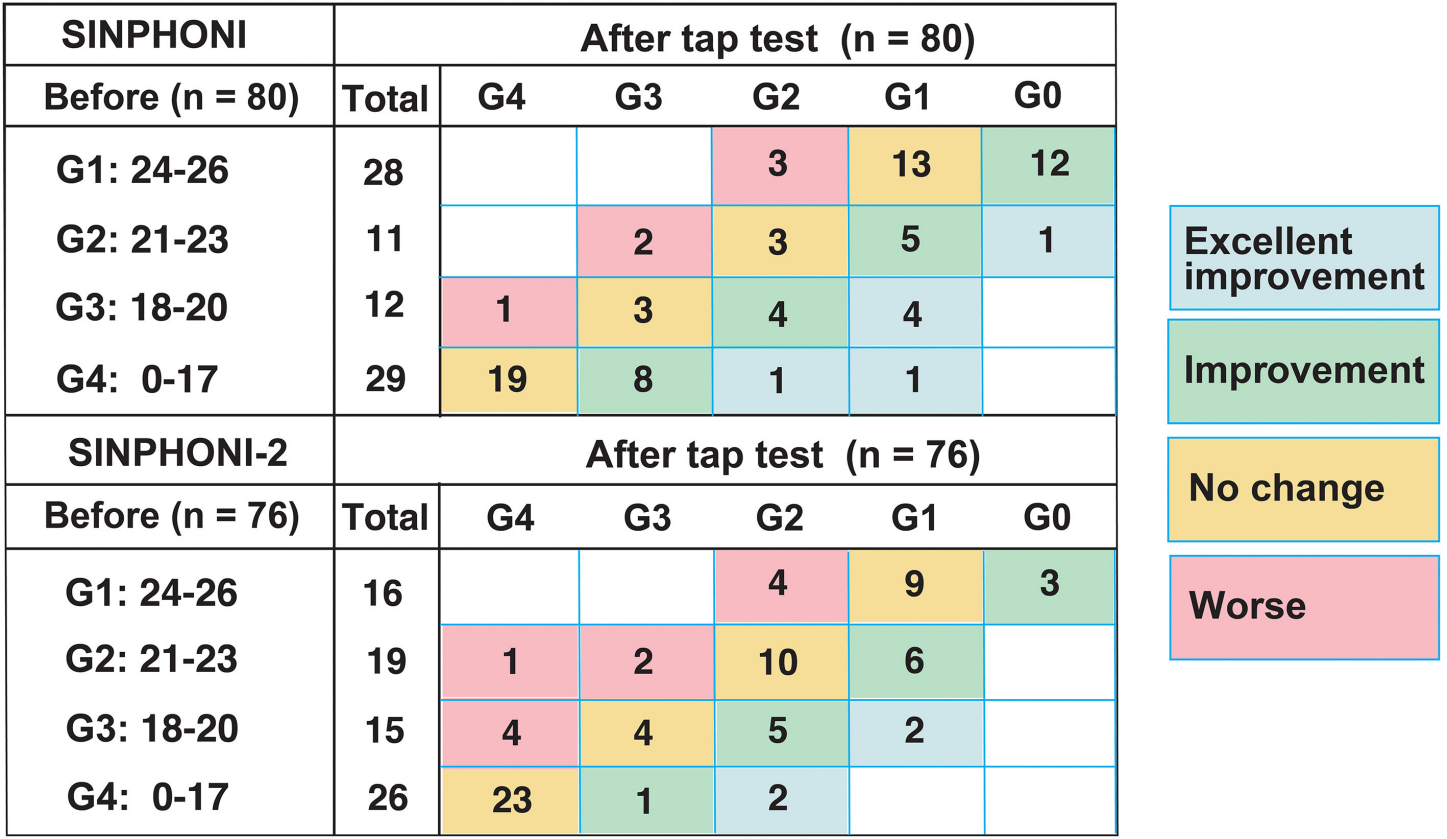
MMSE score changes after the tap test. Distribution of dementia severity grades before and after the tap test, according to MMSE scores in SINPHONI-1 and SINPHONI-2. Numbers in each box indicate the number of patients. Blue, green, and red boxes indicate patients in “excellent improvement,” “improvement,” and “worse” groups. Spearman's rank correlation coefficient and *p*-value between before and after the tap test are ρ: 0.83 (*p* < 0.001) in SINPHONI-1 and ρ: 0.85 (*p* < 0.001) in SINPHONI-2, respectively. G, grade; SINPHONI, study of idiopathic normal pressure hydrocephalus on neurological improvement.

Eighty and seventy-six patients were allocated to the VPS [G1 (*n* = 28), G2 (*n* = 11), G3 (*n* = 12), and G4 (*n* = 29)] and LPS [G1 (*n* = 16, G2 (*n* = 19), G3 (*n* = 15), and G4 (*n* = 26)] cohorts, respectively. The median MMSE score before TT (IQR, 25–75%) in SINPHONI-1 (VPS cohort) and SINPHONI-2 (LPS cohort) were 20 (15–24) points and 20 (16–23) points, respectively (Table 1). When we analyzed changes in each group (G1–G4), 55 of 156 subjects (35.2%) demonstrated improved scores and 17 of 156 subjects (10.9%) demonstrated worsened scores after TT (Figure 3).

**Table 1:** Baseline characteristics before the tap test.

	**Total shunt**	**SINPHONI-1 (VPS)**	**SINPHONI-2 (LPS)**			**Postpone vs. immediate**
		**Total**	**Total**	**Postpone**	**Immediate**	***p*-value**
Total number of patients	156	80	76	35	41	
Men, number (%)	86 (55.1%)	44 (55%)	42 (55.3%)	24 (68.6%)	18 (44%)	
Median age (25–75%), years	76 (72–79)	75 (72–78)	77 (73–79.5)	77 (72.75–80)	77 (73–79)	0.892
MMSE scores, median (25–75%)	20 (15–24)	20 (15–24)	20 (16–23)	19 (14–23)	22 (16.5–24)	0.113
G4: 0–17 points, number (%)	55 (35.3%)	29 (36.3%)	26 (34.2%)	14 (40%)	12 (29.3%)	
G3: 18–20 points, number (%)	27 (17.3%)	12 (15%)	15 (19.7%)	8 (22.9%)	7 (17.1%)	
G2: 21–23 points, number (%)	30 (19.2%)	11 (13.8%)	19 (25%)	9 (25.7%)	10 (24.4%)	
G1: 24–26 points, number (%)	44 (28.2%)	28 (35%)	16 (21.1%)	4 (11.4%)	12 (29.3%)	

*FAB, frontal assessment battery; LPS, lumboperitoneal shunt; MMSE, Mini-Mental State Examination; TMT-A, Trail Making Test A; VPS, ventriculoperitoneal shunt; WAIS-III, Symbol search subtest of the Wechsler Adult Intelligence Scale—Third Edition; ZBI, Zarit Caregiver Burden Interview*.

#### Cerebrospinal Fluid Shunt Placement

**Figure 4:**
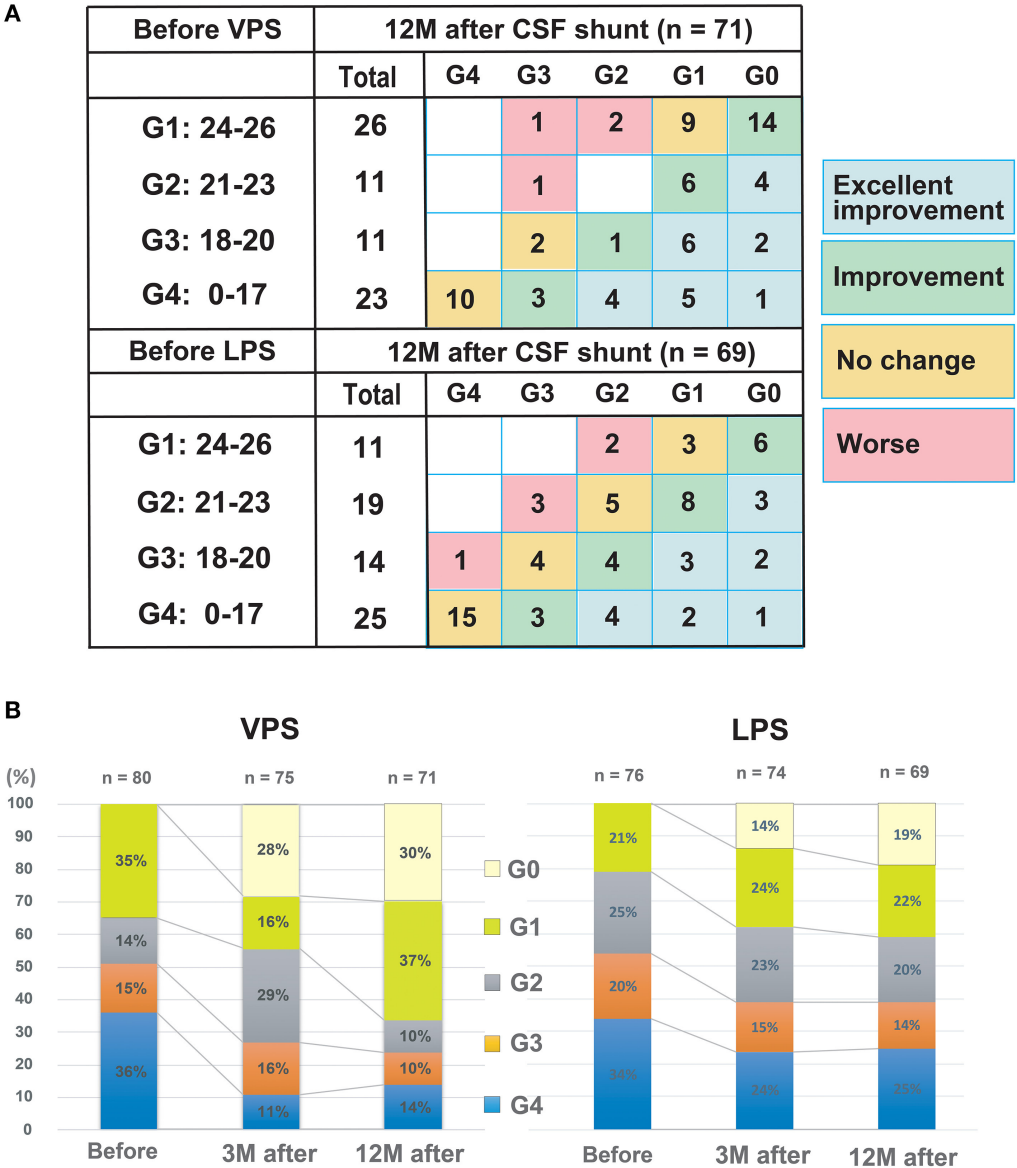
MMSE score changes between before and 12 months after shunting. **(A)** The distribution of preoperative and postoperative grades of dementia severity, according to the MMSE scores. Numbers in each box indicate the number of patients. Spearman's rank correlation coefficient (ρ) and *p*-value between before and 12 months after shunting are ρ: 0.51 (*p* < 0.001) in VPS and ρ: 0.66 (*p* < 0.001) in LPS cohort, respectively. **(B)** Changes after shunt treatment in severity classification by MMSE scores. CSF, cerebrospinal fluid; MMSE, Mini-Mental State Examination; LPS, lumboperitoneal shunt; and VPS, ventriculoperitoneal shunt.

The CSF shunt resulted in a better MMSE score of “improved” or above after 12 months in 81 among 140 subjects (57.8%) (Figure 4A). Twenty-five of 48 subjects (52%) did not fit the G4 classification at 12 months following CSF shunt placement. In other words, approximately half of those in G4 demonstrated improved cognitive function. In G2, improvements were observed in 21 of 30 subjects (70%). Moreover, 18 of 25 subjects (72%) in G3 demonstrated cognitive improvements. In G1, while 20 of 37 subjects (54.1%) revealed improved cognitive function after 12 months, five (13.5%) revealed worsened cognition. The degree of improvement due to the difference in VPS and LPS shunt was not statistically significant.

### Tap Test as a Predictor of Postoperative Mini-Mental State Examination Scores

**Figure 5:**
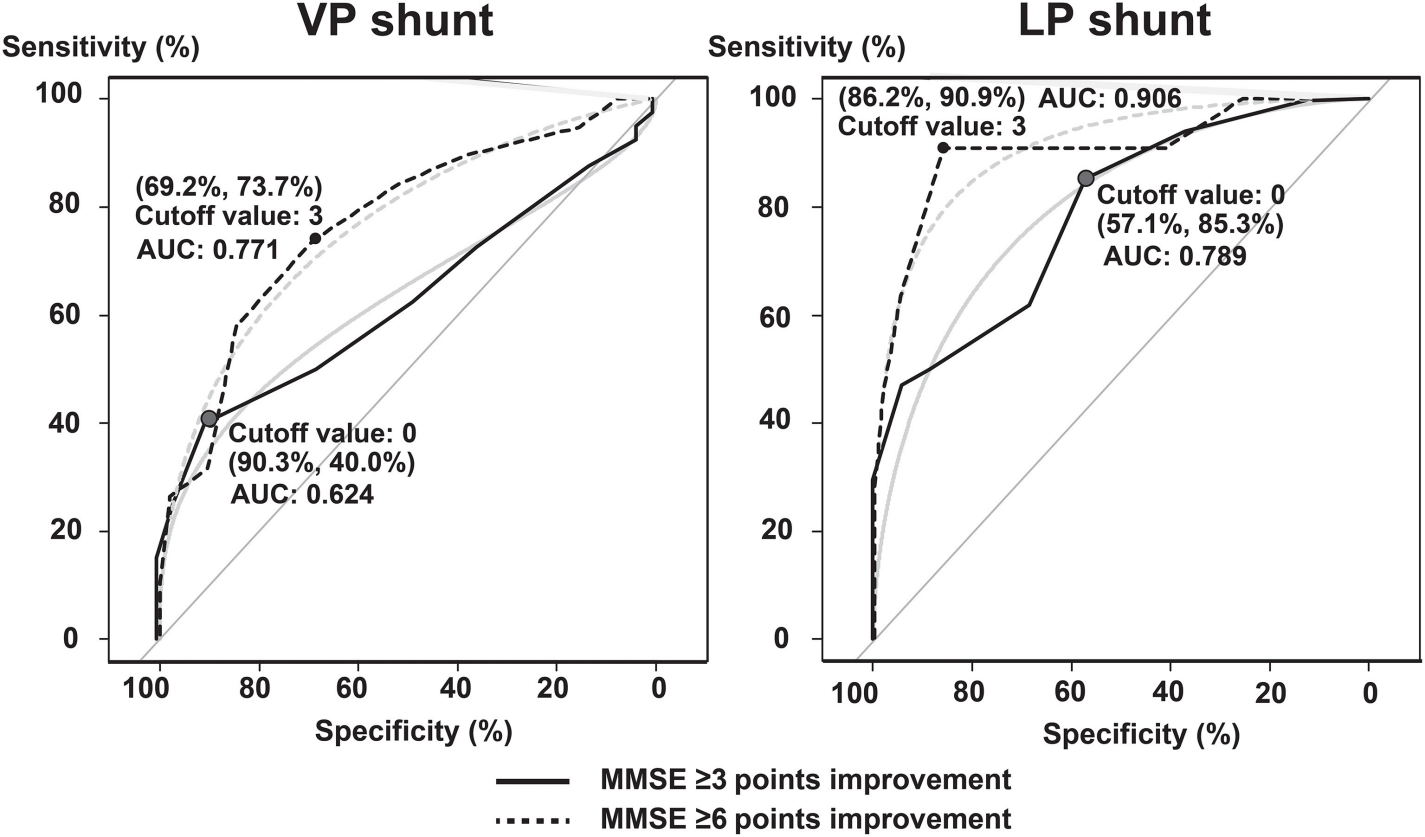
Tap test as predictor of postoperative MMSE scores. The optimal threshold, sensitivity, and specificity of the MMSE score during the tap test to predict MMSE improvement at 12 months after shunting have been analyzed using receiver operating characteristic analysis. The light blue line shows the approximate line. AUC, area under the receiver operating characteristic curve; MMSE, Mini-Mental State Examination; LP, lumboperitoneal; and VP, ventriculoperitoneal.

We performed ROC analysis to evaluate the optimal threshold, sensitivity, and specificity of MMSE scores at the TT to predict improvement at 12 months following shunting. Improvements in MMSE score cutoff values of three points at TT for both VPS and LPS cohorts predicted a likely improvement by six points after treatment. Following shunt intervention, the VPS cohort had a sensitivity, specificity, and AUC of 69.2, 73.7, and 0.771%, respectively, after 12 months. However, the LPS cohort had a sensitivity, specificity, and AUC of 86.2, 90.9, and 0.906%, respectively. Regarding the predicted improvement of three points before TT to 12 months postoperatively, the VPS cohort revealed a sensitivity, specificity, and AUC of 90.3, 40.0, and 0.624%, respectively, for an MMSE score with no change at 1 week after the TT. In contrast, the LPS cohort revealed a sensitivity, specificity, and AUC of 57.1, 85.3, and 0.789%, respectively (Figure 5).

For MMSE scores that improved by ≥3 points after TT, the possibility of an improvement by 6 points after 12 months following the intervention was A-OR 7.77 (95% CI: 2.22–27.2) and M-OR 6.30 (1.74–22.8) times higher in the VPS cohort. Conversely, the possibility was A-OR 62.3 (6.96–557.5) and M-OR 59.6 (6.64–535.6) times higher in the LPS cohort (Table 2).

**Table 2:** The odds ratio for improvement of scores on MMSE after shunt surgery.

**Before shunt**	**After shunt**									
**At tap test**	**Worse or <6 improved**	**≥6 scores improved**	**Sensitivity (95% CIs)**	**Specificity (95% CIs)**	**A-OR^†^**	**(95% CIs)**	***p*** **-value**	**M-OR** ^**§**^	**(95% CIs)**	***p*** **-value**
**12 months after VPS in SINPHONI-1 (*****n*** **= 71)**										
Worse or <3 improved	36	5	0.69	0.74	Reference		Reference			
≥3 scores improved	16	14	(0.62–0.74)	(0.55–0.87)	7.77	(2.22–27.2)	^***^ <0.001	6.30	(1.74–22.8)	^**^0.004
**12 months after LPS in SINPHONI-2 (*****n*** **= 69)**										
Worse or <3 improved	50	1	0.86	0.91	Reference		Reference			
≥3 scores improved	8	10	(0.82–0.88)	(0.66–0.98)	62.3	(6.96–557.5)	^***^ <0.001	59.6	(6.64–535.6)	^***^ <0.001
**At tap test**	**Worse or <3 improved**	**≥3 scores improved**	**Sensitivity**	**Specificity**	**A-OR^†^**	**95% CIs**	***p*** **-Value**	**M-OR** ^**§**^	**95% CIs**	***p*** **-Value**
**12 months after VPS in SINPHONI-1 (*****n*** **= 71)**										
Worse	4	5	0.13	0.88	Reference		Reference			
≥0 scores improved	27	35	(0.06–0.21)	(0.82–0.94)	1.06	(0.26–4.39)	0.93	1.18	(0.27–5.09)	0.82
**12 months after LPS in SINPHONI-2 (*****n*** **= 69)**										
Worse	20	5	0.57	0.85	Reference		Reference			
≥0 scores improved	15	29	(0.46–0.65)	(0.74–0.93)	7.51	(2.33–24.2)	^***^ <0.001	7.54	(2.91–24.8)	^***^ <0.001

** *p < 0.01*,

*** *p < 0.001*.

*A-OR^†^, age-adjusted odds ratio; M-OR§, multivariate-adjusted odds ratio, which was adjusted by age and MMSE score before shunt surgery; 95% Cis, 95% confidence intervals; LPS, lumboperitoneal shunt; MMSE, Mini-Mental State Examination; VPS, ventriculoperitoneal shunt*.

For no change in MMSE scores by the TT, the possibility of an improvement by three points by 12 months following the intervention was A-OR 1.06 (0.26–4.39) and M-OR 1.18 (0.27–5.09) in the VPS cohort, compared with A-OR 7.51 (2.33–24.2) and M-OR 7.54 (2.91–24.8) in the LPS cohort. For cutoff values (MMSE score 0 improvement), the prediction was effective only for the LPS cohort (Table 2).

## Discussion

The iNPH syndrome has emerged as a more treatable gait disorder than a treatable form of dementia (27). There are few reports of objective neuropsychological assessment methods to predict the effect of shunt surgery (28–30). We confirmed that cognitive function improves with iNPH using objective measures of MMSE scores rather than subjective measures such as the Grading Scale and showed that a TT can predict the effect of cognitive function improvement after shunt surgery. Other cognitive function tests, such as WAIS-III, TMT-A, and ZBI, also showed statistically significant improvement at 12 months after shunting compared with preoperative levels (Supplementary Table 2). These results indicate that shunting iNPH patients with cognitive impairment has a role to play in reducing the burden on caregivers, as a clear improvement in cognitive function was demonstrated.

Herein, 57.8% of iNPH patients with cognitive impairment demonstrated “cognitive improvement” (improvement by one or more grades) at 12 months after shunt placement. However, there was a difference in the improvement between G2 (70%), G3 (72%) on one side, and G1 (54%), G4 (48%). Patients with moderate cognitive decline (G2 and G3), whose preoperative MMSE scores ranged from 18 to 23 points, showed a significant improvement in cognitive function after shunt intervention. Following shunt placement, about half of the patients in the G1 group with MCI revealed improved cognition compared with the normal range. This was partially because elderly people are the target base of iNPH and partly because of a ceiling effect. Also, improvement was less in patients with severe cognitive disturbance (G4 group) who had MMSE scores ≤17 (Figure 4B). The finding that iNPH patients with more severe disability do not improve after surgery is similar to that reported for gait disturbance (14). It may indicate that cognitive dysfunction may not improve when reaching more severe stages (27, 31–34).

Cognitive function changes appear later compared with other iNPH symptoms, such as gait disturbance (14). Considering that the MMSE score changes due to TT are not considerably large, it might be insufficient to evaluate cognitive function during a time period of 7 days after TT (30, 35, 36). However, MMSE score changes between “before TT” and 7 days after are useful in predicting the level of improvement at 12 months after shunt surgery. Following TT, MMSE score change groups with improvement or even without change groups tended to continue their increase 12 months after CSF shunting. The cutoff value (MMSE score <3 or ≥3) was a valid predictor of an improvement of ≥6 points in MMSE after both VPS and LPS. Practically, if a patient had responded to TT, a better effect of treatment with LPS can be expected, and in an improvement in MMSE score changes <3 points only, obtained by TT, an improvement of MMSE ≥6 after the shunt cannot be expected. The LPS group results—in which, even after intervention, CSF can be drained from the lumbar subarachnoid space—were highly correlated with the changes after TT. It became clear that if there was no improvement in MMSE in the TT, there would be no improvement in MMSE scores after LPS intervention and, therefore, should be avoided owing to the fact that it worsens cognitive function.

On the other hand, we can speculate that in VPS, cognitive function improvement was not reflected by the TT result for patients with iNPH where the CSF flow to the lumbar subarachnoid space might be affected, e.g., due to spondylosis, but shunt effectiveness can still be obtained. These trends, such as the LPS being more reflective of TT results, are similar to previous reports assessing gait disturbance after TT (24). We supposed that the reason for these results might be in the inclusion criteria of the SINPHONI-1 and 2 study, which were based on patients with disproportionately enlarged subarachnoid space hydrocephalus (DESH) as an imaging finding (20). In patients with probable iNPH and DESH findings, indications for shunting do not require an improvement in MMSE of three points or more on the TT, as indicated in the first and second Japanese iNPH guidelines (4, 5, 9), but rather, any improvement in MMSE of three points or more on the TT is a predictive marker for an additional improvement of six points or more after shunting.

In an earlier report, Miyajima et al. reported that there was no significant difference between the two methods, using data from SINPHONI-1 and SINPHONI-2 trials (23). No randomized trials have directly compared the aforementioned surgical techniques. It is necessary to discuss the optimal shunt treatment method, either VPS or LPS, for iNPH patients based on the individual patient situation.

This study has some limitations. The number of participants who were recruited was small. We conducted a multicenter cooperative study and entrusted patient selection to each facility. Despite the inclusion of patients meeting the clinical criteria, surgeons may have excluded cases complicated by neurodegenerative diseases, such as Alzheimer's disease or dementia with Lewy bodies, and a considerable number of them are expected to have iNPH, causing a potential selection bias.

Furthermore, in this study, short-term improvement in cognitive function was achieved with shunting. However, elucidating cognitive changes over a longer-term course or in cases of iNPH with comorbid diseases is a challenge for iNPH treatment selection. It has been reported that CSF biomarkers are useful to differentiate patients with iNPH with comorbid neurodegenerative diseases and that patients with Alzheimer's disease are less likely to have their cognitive function improved (37–44). We believe that in the present cohort, the multicenter study (SINPHONI-1 and SINPHONI-2) may include selection bias that recruited iNPH without comorbidities as much as possible.

Currently, all iNPH severity classifications are based on preoperative symptoms. The necessity of revising severity classifications that predict the degree of improvement from shunting for intervention selection is another challenge of the future.

In conclusion, CSF shunting contributes to an improvement in cognitive function for patients with iNPH. Furthermore, MMSE score evaluation at the TT can sensitively predict an improvement in postoperative MMSE scores after LPS intervention.

## Data Availability Statement

The data that support the findings of this study are available on reasonable request from the corresponding author, MN. The data are not publicly available since they include information that could compromise the privacy of the research participants.

## Ethics Statement

The studies involving human participants were reviewed and approved by the Ethics Committee of Juntendo University. The patients/participants provided their written informed consent to participate in this study. Written informed consent was obtained from the individual(s) for the publication of any potentially identifiable images or data included in this article.

## Co-Investigators of SINPHONI-2

Masaaki Hashimoto, MD, PhD (Noto General Hospital, Advisory Committee); Hideki Origasa, MD, PhD (University of Toyama, study statistician); Haruko Yamamoto, MD, PhD (National Cerebral and Cardiovascular Center, Independent Data and Safety Monitoring Committee); Hajime Arai, MD, PhD (Juntendo University, Advisory Committee); Koreaki Mori, MD, PhD (Kochi Medical School, Independent Data and Safety Monitoring Committee); Shigenobu Nakamura, MD, PhD (Clinical Research Center, Independent Data and Safety Monitoring Committee); Tamotsu Miki, MD, PhD (Tokyo Medical University, Independent Data and Safety Monitoring Committee); Kazunari Ishii, MD, PhD (Kinki University School of Medicine, director of Imaging Committee); Hiroji Miyake, MD, PhD (Kyoritsu Neurosurgical Hospital, Advisory Committee); Nobumasa Kuwana, MD, PhD (Tokyo Kyosai Hospital, site investigator); Naoyuki Samejima, MD, PhD (Tokyo Kyosai Hospital, site investigator); Daisuke Kita, MD, PhD (Noto General Hospital, site investigator); Takahiko Tokuda, MD, PhD (Kyoto Prefectural University of Medicine, site investigator); Mitsuhito Mase (Nagoya City University Graduate School of Medical Sciences, site investigator); Satoru Mori, MD, PhD (University of Shiga Prefecture, Advisory Committee); Yoshinaga Kajimoto, MD, PhD (Osaka Medical Collage, site investigator); Teiji Nakayama, MD, PhD (Hamamatsu Medical Centre, site investigator); Osamu Hirai, MD, PhD (Shinko Hospital, Advisory Committee); Masatoshi Takeda, MD, PhD (Osaka University Graduate School of Medicine, Advisory Committee); Chia-Cheng Chang, MD, PhD (Yokohama Minami Kyosai Hospital, site investigator); Isao Date, MD, PhD (Okayama University Graduate School of Medicine, site investigator); Masahiro Kameda, MD, PhD (Okayama University Graduate School of Medicine, site investigator); Takaharu Okada, MD, PhD (Tama-Hokubu Medical Center, site investigator); Junichiro Hamada, MD, PhD (Kanazawa University Graduate School of Medicine, site investigator); Mitsuya Watanabe, MD, PhD (Tama-Nanbu Regional Hospital, site investigator); Mitsunobu Kaijima, MD, PhD (Megumino Hospital, site investigator); Souichi Sunada, MD, PhD (Tsudanuma Central General Hospital, site investigator); Yoshihumi Hirata, MD, PhD (Kumamoto Takumadai Hospital, Advisory Committee).

## Author Contributions

MI and EM conceived and coordinated the study and chaired the steering committee. MN prepared the first draft of the manuscript. All authors were members of the steering committee and designed and wrote the study protocol with input from all listed members of the study advisory board, involved in the interpretation and/or presentation of the data, reviewed and revised the initial draft and subsequent versions of the report, and approved the submitted version, listed investigators contributed to the patient enrollment, the steering committee, independent data, and safety monitoring committee monitored the study.

## Funding

This work was supported by the Japan Society for the Promotion of Science under Grants-in-Aid for Scientific Research (grant numbers 16KK0187, 17K10908, 18H02916, and 20K09398) and the Japan Agency for Medical Research and Development (grant number AMED JP20dm0207073).

## Conflict of Interest

This investigator-initiated study was supported in part by Johnson & Johnson K.K. and Nihon Medi-Physics Co. Ltd. This work was also supported in part by the Ministry of Health, Labor, and Welfare of Japan (2014-Nanchi-General-052), and by Grants-in-Aid for Scientific Research (grant numbers 16KK0187, 17K10908, 18H02916, and 20K09398) from the Japan Society for the Promotion of Science. The funders were not involved in the study design, collection, analysis, interpretation of data, the writing of this article or the decision to submit it for publication. MN has received speakers' honoraria from Johnson & Johnson K.K., Janssen Pharmaceutical K.K., and Medtronic, Inc. SY received speaker honoraria from Johnson & Johnson, K.K. and Medtronic, Inc. MM has received speaker's honoraria from Johnson & Johnson K.K., Nihon Medi-Physics Co. Ltd., Medtronic, Inc., Eisai Inc., and Daiichi-Sankyo Co., Ltd. HK has received speakers' honoraria from Johnson & Johnson K.K., Nihon Medi Physics Co. Ltd., Medtronic, Inc., Ono Pharmaceutical Co., Ltd, Pfizer Inc., Eisai Inc., Daiichi-Sankyo Co., Ltd., Novartis Pharma K.K., Janssen Pharmaceutical K.K., and Takeda Pharmaceutical Co., Ltd. EM has received research grants from Eisai Co. Ltd. and Fuji Film RI, consulting fees from Lundbeck, and speaker's honoraria from Johnson & Johnson K.K., Nihon Medi-Physics Co. Ltd., Medtronic, Inc., Eisai, Co., Ltd., Daiichi Sankyo Co., Ltd., Janssen Pharmaceutical K.K., Novartis Pharma K.K., and Ono Pharmaceutical Co. Ltd. MI received speaker honoraria from Johnson & Johnson, K.K. and Medtronic, Inc. The remaining authors declare that the research was conducted in the absence of any commercial or financial relationships that could be construed as a potential conflict of interest.

## Publisher's Note

All claims expressed in this article are solely those of the authors and do not necessarily represent those of their affiliated organizations, or those of the publisher, the editors and the reviewers. Any product that may be evaluated in this article, or claim that may be made by its manufacturer, is not guaranteed or endorsed by the publisher.
