# Supplementary material for: Tap Test Can Predict Cognitive Improvement in Patients With iNPH—Results From the Multicenter Prospective Studies SINPHONI-1 and –2
Source: Front Neurol. 2021 Nov 2;12:769216. doi: 10.3389/fneur.2021.769216 (PMC8593336; doi:10.3389/fneur.2021.769216)
Supplement: Supplementary file 1 [file Data_Sheet_1.docx]

**Supplementary table 1**

**Neuropsychological test’s results of cognitive impairment with iNPH**

|  | **SINPHONI-2 (LPS)** | | **postpone vs immediate** |
| --- | --- | --- | --- |
|  | **Postpone, median (25%–75%),** | **Immediate, median (25%–75%),** | ***p*-value** |
| **MMSE scores, before** | 19 (14–23) | 22 (16.5–24) | 0.113 |
| **7 days after a tap test** | 21 (14–24) | 21 (17–24) | 0.481 |
| **12 months after shunt** | 21 (14.5–24) | 23 (19–26.75) | 0.059 |
| **FAB scores, before** | 9.5 (7.25–12) | 10 (7–11) | 0.665 |
| **12 months after shunt** | 11 (7.5–13) | 10 (7–14) | 0.713 |
| **WAIS-Ⅲ scores before** | 9 (3–14) | 7 (2–12) | 0.384 |
| **12 months after shunt** | 7.5 (2–15.25) | 9 (3–15.5) | 0.649 |
| **TMT-A (sec), before** | 113 (77.9–165) | 105 (72.5–154.5) | 0.964 |
| **12 months after shunt** | 98 (66–195) | 85 (51.97–170.75) | 0.342 |
| **ZBI scores, before** | 36 (21–49) | 24.5 (12.25–45.75) | 0.112 |
| **12 months after shunt** | 23.5 (9–45.5) | 16 (7.75–33.5) | 0.276 |

**Abbreviations**: FAB, Frontal Assessment Battery; TMT-A, Trail Making Test-A; WAIS-III, Symbol search subtest of the Wechsler Adult Intelligence Scale–Third Edition; ZBI, Zarit Caregiver Burden Interview; CSF, cerebrospinal fluid; MMSE, Mini-Mental State Examination; LPS, lumboperitoneal shunt.

**Supplementary table 2**

**Cognitive change iNPH patients with MMSE scores ≤ 26 points in SINPHONI-2**

|  | **Before,**  **(n=76)** | **Tap test after 7 days,**  **(n=76))** | **3 months after shunt, (n=72)** | **12 months after shunt, (n=69)** | ***p*-value**  p1: before vs 7d, p2: before vs 3M, p3: before vs 12M |
| --- | --- | --- | --- | --- | --- |
| **Neuropsychological test, median (25%–75%)** | | | | |  |
| **MMSE scores** | 20 (16–23) | 21 (16–24) | 22 (17–25) | 22 (17–25.5) | p1: 0.23,  p2: <0.001***,  p3: <0.001*** |
| **FAB scores** | 9 (6–12) | 10 (7–12) | 11 (8-14) | 10 (7–13) | p1: 0.049*,  p2: 0.005**,  p3: 0.093 |
| **WAIS-Ⅲ scores** | 7 (2.75–12.25) | 8(2–14) | 8.5 (4–15.75) | 8 (3–15) | p1: 0.143,  p2: 0.002**,  p3: 0.003** |
| **TMT-A (sec)** | 107.5 (70–175.5) | 107 (72.25–184.5) | 97.5 (72.5–201.9) | 88 (62–178) | p1: 0.548,  p2: 0.137,  p3: 0.009** |
| **ZBI scores** | 26.5 (13–47) | N/A | 22 (9–38) | 19 (8–39.5) | p2: <0.001***,  p3: <0.001*** |

*p<0.05, **p<0.01, ***p<0.001

**Abbreviations:**

MMSE, Mini-Mental State Examination; FAB, Frontal Assessment Battery; TMT-A, Trail Making Test-A; WAIS-III, Symbol search subtest of the Wechsler Adult Intelligence Scale–Third Edition; ZBI, Zarit Caregiver Burden Interview.
